# Person-centered care planning in Medicaid 1915(c) Home and Community Based Service (HCBS) waivers for older adults

**DOI:** 10.1093/geroni/igaf122.3034

**Published:** 2025-12-31

**Authors:** Natalie Turner

**Affiliations:** University of Washington, Seattle, Washington, United States

## Abstract

Person-centered care planning is an approach to service coordination that prioritizes an individual’s goals, needs, preferences, and desires. Over the past decade, the Administration for Community Living and the Centers for Medicare & Medicaid Services have worked to advance person-centered planning within home and community-based services (HCBS). 1915(c) waivers can offer insights into how states apply these principles in care policy. This qualitative study examined how person-centered care planning was described in 63 1915(c) waivers for older adults using the Frameworks Approach, a qualitative method designed for applied policy research. Codes were developed deductively based on waiver application questions and applied to select appendices detailing care planning processes. Data were summarized into matrices and analyzed to identify four key themes: (1) Care planning led and owned by participants; (2) Holistic approaches to care planning; (3) Individualization of services; and (4) Care plans as living, adapting documents. Waivers emphasized participant-driven decision-making, with language such as “Member choice drives all decisions.” Care planning was also holistic, considering not only health and functional needs but also social life, community engagement, and other enriching activities voiced by participants. Plans required documentation of individual preferences on service delivery, including schedules and desired activities. Finally, care plans were expected to evolve alongside participants’ changing needs. These findings highlight how person-centered care planning is operationalized in care policy and underscore the importance of individual agency in HCBS. Future research should examine how the operationalization of person-centered planning compares to its implementation to assess alignment and effectiveness.

